# A case of hypopituitarism with pancytopenia cured by corticosteroid and thyroid hormone replacement therapy 

**DOI:** 10.1530/EDM-24-0119

**Published:** 2025-07-21

**Authors:** Violeta Mladenovic, Radica Zivkovic Zaric, Snezana Sretenovic, Dragana Bubanja, Zeljko Ivosevic, Nebojsa Igrutinovic, Jelena Nesic, Predrag Djurdjevic

**Affiliations:** ^1^ Faculty of Medical Sciences, University of Kragujevac, Kragujevac, Serbia; ^2^ University Clinical Center Kragujevac, Department of Internal Medicine, Kragujevac, Serbia; ^3^ University Clinical Centar Kragujevac, Department for Clinical Pharmacology, Kragujevac, Serbia

**Keywords:** pancytopenia, hypopituitarism, Sheehan’s syndrome

## Abstract

**Summary:**

Pancytopenia associated with hypopituitarism has been reported in the literature as a rare occurrence limited to isolated case reports, predominantly associated with Sheehan syndrome. We present the case of a 31-year-old woman who showed hematological features of pancytopenia and normal cellularity of bone marrow. Hematological investigation disclosed no other cause for pancytopenia. Her physical findings (generalized weakness, slow speech and no pubic or axillary hair) and history of a previous massive postpartum hemorrhage suggested Sheehan’s syndrome, and the pituitary hormonal studies revealed panhypopituitarism. Her blood cell accounts were completely recovered after 5 months of glucocorticoid and thyroxine replacement therapy. We hereby report our experience of a cure of pancytopenia and the normal marrow originating from hypopituitarism after corticosteroid and thyroid hormone replacement therapy.

**Learning points:**

- Sheehan’s syndrome correlates with postpartum hemorrhage and causes pancytopenia.
- It could be successfully treated with glucocorticoid.
- It could be successfully treated with thyroxine replacement therapy.

## Background

Sheehan (1), in 1937, described the syndrome of postpartum hemorrhage (PPH), pituitary necrosis, and lactation failure as one of the most important causes of hypopituitarism. However, clinically, such patients often present late with severe hypopituitarism and circulatory collapse (2, 3). Enlargement of the pituitary gland, small sella size, disseminated intravascular coagulation, and autoimmunity have been suggested to play a role in the pathogenesis of Sheehan’s syndrome (SS) in women who suffer from severe PPH (3).

Pancytopenia is a rare hematological manifestation of a hormonal abnormality. Still, hematologists need to be aware of panhypopituitarism as a differential diagnosis when women showing features of hypopituitarism present with pancytopenia because it can be recovered with adequate hormone replacement. Normocytic and normochromic anemia can occur in patients with hypopituitarism. Furthermore, a few complete bone marrow aplasia cases, including pancytopenia, have been reported (4).

## Case presentation

We describe a 31-year-old woman who presented with normocytic and normochromic anemia, leucopenia, and severe thrombocytopenia. Namely, a patient was admitted to our hospital after an incidentally found pancytopenia on a routine blood cell count examination. Three years ago, after her third delivery, she complained of generalized weakness and a tingling sensation on both upper and lower extremities. After careful history taking, it was found that she had experienced an episode of PPH in the course of her third delivery. During the performed physical examination, we revealed that her speech was slow and she had no pubic or axillary hair. She also complained about generalized weakness. There was no family history of similar symptoms and no history of trauma. Our patient underwent a series of laboratory examinations and a bone marrow examination to determine the cause of her hematologic abnormalities.

## Investigation

The laboratory findings were a total leukocyte count of 2.1 × 10^9^/L with severe neutropenia (0.4 × 10^9^/L) while the platelet count was 19 × 10^9^/L (Table 1). The hemoglobin level was 78 g/L, reticulocyte count was 0.5% with mean cell volume (MCV) 85.8 fl, mean cell hemoglobin (MCH) 29.1 pg, and mean cell hemoglobin concentration (MCHC) 340 g/dL. Fasting blood samples were obtained for liver and kidney functions, electrolytes, and glucose. Baseline clinical and hematological parameters were in the reference range, summarized in Table 2. The serum iron concentration was 18.1 mg/dL (reference range (RR): 7.3–32.2 mg/dL), total iron binding capacity (TIBC) was 56 mg/dL (RR: 48–56 mg/dL) and ferritin level was 29 (RR: 20–300 μg/L). All determined tumour markers (alpha-fetoprotein (AFP), carcinoembryonic antigen (CEA), CA 19-9, CA 125-II) were in the reference ranges (Table 3). The immunological analysis was also normal (Table 4). The peripheral blood smear did not show any morphological abnormalities in the peripheral blood cells. Serial stool and urine examinations which were done to exclude the possibility of chronic gastrointestinal and urogenital bleeding were negative. A performed bone marrow examination showed a mildly hypercellular marrow with a markedly increased granulopoiesis followed by moderately decreased erythropoiesis. The number of megakaryocytes was normal with mild nuclear hyperploidy. There were no features for lymphoproliferative, myeloproliferative, and metabolic diseases, and metastatic cancer. Under the suspicion of the SS, a series of pituitary and end-organ hormone tests were performed (Table 5). The criteria for the diagnosis of SS were as follows: i) typical obstetric history of intrapartum or postpartum bleeding, ii) hypotension or shock, iii) agalactia, iv) failure to resume regular menses after delivery, v) hypopituitarism, and vi) empty sella on computed tomography (CT) or magnetic resonance imaging (MRI) (5).

**Table 1:** Trends of the hematological features before and after the initiation of the hormonal replacement therapy.

Parameter	At admission	After 2 months	After 5 months
Leucocytes (×10^9^/L)	2.1	3.2	4.5
Platelets (×10^9^/L)	19	102	163
Hemoglobin (g/L)	78	90	121

**Table 2:** Baseline biochemical analysis.

Parameter	Value	Reference range
Potassium (mmol/L)	4.8	3.5–5.3
Sodium (mmol/L)	141	137–147
Calcium (mmol/L)	2.39	2.2–2.65
Phosphate (mmol/L)	1.16	0.8–1.6
Proteins (g/L)	74	64–83
Albumins (g/L)	42	35–52
Urea (mmol/L)	5.1	3.0–8.0
Creatinine (μmol/L)	65	49–106
Acidum uricum (μmol/L)	206	156–428
Total cholesterol (mmol/L)	5.25	3.1–5.2
HDL cholesterol (mmol/L)	1.78	1.1–2.5
LDL cholesterol (mmol/L)	3.69	0.1–3.5
Triglyceride (mmol/L)	1.7	0.1–1.7
SGOT (U/L)	31	1–40
SGPT (U/L)	13	1–40
Gama GT (U/L)	37	7–50
Bilirubin (μmol/L)	8.0	5–21
LDH (U/L)	323	220–450

SGOT, serum glutamic oxaloacetic transaminase; SGPT, serum glutamic pyruvate transaminase; gamma-GT, glutamate transferase; LDH, lactate dehydrogenase.

**Table 3:** Tumour markers.

Tumor markers	Value	Reference range
CEA (ng/mL)	2.4	3 – 5
CA 19-9 (U/mL)	<3.0	<37
CA 125-II (U/mL)	12.37	<35
AFP	4.33	

CEA, carcinoembryonic antigen; AFP, alpha fetoprotein.

**Table 4:** Immunology parameters.

Parameter	Value	Reference range
IgA (g/L)	1.79	0.7–4.0
IgG (g/L)	16.91	7–16
IgM (g/L)	1.45	0.4–2.3
C3 (g/L)	0.92	0.9–1.8
C4 (g/L)	0.15	0.1–0.4
RF	2.6	0–30
IC	0.47	<0.5
ANA	Negative	
AMA	Negative	

RF, rheumatic factor; IC, circulating immune complexes; ANA, antinuclear antibodies; AMA, antimitochondrial antibodies.

**Table 5:** Results of pituitary and end organ hormone tests. Abnormal values are presented in bold.

Hormone	Test value	Reference range
FSH (mIU/mL)	**3.42**	17–95
LH (mIU/mL)	**0.91**	8–33
17-Beta-estradiol (pg/mL)	9.74	<58
Progesterone (ng/mL)	0.39	<0.41
Testosterone	**<0.1**	0.1–0.9
Serum cortisol (nmol/L)		
8 AM	**121**	154–638
8 PM	**74.7**	80–388
12 PM	**49.9**	
Prolactin (µIU/mL)		
8 PM	178	130–700
11 PM	175	
1 PM	163	
ACTH (pg/mL)	24.8	<46.0
IGF-1 (ng/mL)	**<25.0**	115–307
fT4 (pg/mL)	**4.9**	7–18
TSH (mIU/L)	2.8	0.25–4
Thyroglobulin (ng/mL)	0.2	<50
Anti Tg Ab (IU/mL)	23.4	0–30
Anti TPO Ab (IU/mL)	37.6	<50
PTH (pg/mL)	7.3	<70
Calcitonin (pg/mL)	<1	<70

FSH, follicle-stimulating hormone; LH, luteinizing hormone; ACTH, adrenocorticotropic hormone; IGF-1, insulin-like growth factor; TSH, thyroid-stimulating hormone; fT4, free thyroxine; anti TPO Ab, anti thyroid peroxydase antibodies; PTH, parathyriod hormone.

Our examination showed the results compatible with a status of panhypopituitarism. The patient’s physical findings, history of a previous massive PPH, and hormonal status suggested the SS. The pituitary function was assessed by determination of basal serum free thyroxine (fT4), thyroid-stimulating hormone (TSH), the daily profile of cortisol, the daily profile of prolactin, luteinizing hormone (LH), follicle-stimulating hormone (FSH), and 17-estradiol measurements using commercial kits. All hormone assays were batched together (5). Pituitary hormone studies were compatible with a status of primary pituitary insufficiency. We performed a Synacthen stimulation test (ACTH, Table 6) and an insulin tolerance test (Table 7). We also performed T1- and T2-weighted MRI. The patient’s pituitary gland was hypoplastic and sella turcica was filled with the cerebrospinal fluid (empty sella). Diagnosis of SS in this patient was based on the history of postpartum hemorrhage, lactation failure, secondary amenorrhea, hormonal evidence of varying degrees of hypopituitarism, and empty sella on pituitary imaging. Hormonal estimations revealed lower levels of FT4, TSH, LH, FSH, PRL, GH, and cortisol.

**Table 6:** Short ACTH test (25ij iv).

Time, minutes	Serum cortisol, nmol/L	Reference range
0	318	154–638
30	366	154–638
60	474	154–638

**Table 7:** Insulin tolerance test (0.15 ij Actrapid HM /kg i.v.).

Time	Glycemia (mmol/L)	Serum cortisol (nmol/L)	Insulin (μIU/mL)	Prolactin (μmol/L)
8 h	3.6	150	11.2	278
8 h 15 min	2.9	153	395	273
8 h 30 min	1.5	167	90.2	297
8 h 45 min	10.4	139	833	201
9 h	15.6	141	758	254
9 h 30 min	10.4	141	232	253
10 h	10.8	139	85	254

## Treatment

The patient received three doses of deplasmatized erythrocytes, 3 liters of isotonic solutions (the first 3 days), and then, according to the hemodynamic parameters, parenteral intake was reduced and liquid intake was started per os. We began to treat our patient with platelet and erythrocyte transfusion, granulocyte colony-stimulating factor (CSF-G), antibiotics, antifungal and antiviral drugs.

Endocrinological treatment consisted of glucocorticoid, thyroxine and estrogen and progesterone replacement therapy.

The patient had been followed up routinely with a complete blood count examination once a month. After 5 months of glucocorticoid and thyroxine replacement therapy, her pancytopenia recovered completely. The leukocyte count was 4.5 × 10^9^/L, the hemoglobin level was 121 g/L, and the platelet count was 163 × 10^9^/L. Table 1 shows the trends of the hematological features before and after initiating the hormonal replacement therapy. A follow-up bone marrow biopsy revealed normal hematopoietic cells with regenerating features. Routine biochemical tests showed that the serum sodium and potassium levels were also normal.

## Outcome and follow-up

Her body weight increased from 39.0 to 49.0 kg. In summary, from the above results, it was concluded that patients’ pancytopenia and hypoplastic bone marrow originated from the previous longstanding hypopituitarism.

## Discussion

SS is the occurrence of pituitary necrosis and hypopituitarism following massive bleeding after normal delivery, usually preceded by postpartum hemorrhage and hypotension. Characteristically, this occurs after delivery, in which the mother loses a significant amount of blood. This blood loss makes the pituitary gland incapable of secreting hormones. Higher pituitary capacity and cell sum occur in pregnant women weeks before delivery. This increase is produced basically by hyperplasia of prolactin-producing cells (lactotrophs) and hyperplasia of other cells in the anterior pituitary gland. The blood supply that feeds the anterior pituitary does not increase, even though hyperplasia leads to increased nutritional and metabolic requirements by the anterior pituitary gland. Not all Sheehan’s develop hematological complications. This is because the severity and specific pattern of pituitary hormone deficiency, along with individual variations in bone marrow response and other factors, influence the development of these complications. The treatment of Sheehan syndrome is the addition of deficient hormones. Hypothyroidism can be cured with levothyroxine or liothyronine. Lack of cortisol can be treated with prednisone or hydrocortisone. Gonadotropin absence should be cured with estrogen (6, 7). Various hematological abnormalities, including anemia and pancytopenia, have been described in these patients (7, 8). SS has a varying clinical presentation; most patients have complete anterior pituitary failure (8). Although a small number of patients with SS may have an abrupt onset of severe hypopituitarism immediately after delivery, most patients have mild disease and they live undiagnosed and untreated for a long time inappropriately (6). Most patients present with insidious onset lethargy, weakness, and loss of pubic and axillary hair. However, some patients present with acute adrenal insufficiency, hypotension, and hypoglycemia (6). It may result in partial hypopituitarism or panhypopituitarism. Most of these patients have the empty sella on CT or MRI (4).

Sheehan’s description of the syndrome promoted the belief that PPH leads to necrosis of the hyperplastic pituitary gland of pregnancy with consecutive hypopituitarism and failure of lactation (1). However, some patients have slow clinical progression, which suggests factors other than ischemia in its pathogenesis (4). It also seems that SS has hematological consequences, to which little attention is paid because of their rarity. Anemia is well-recognized as a feature of hypopituitarism (8). Pathogenesis of anemia in panhypopituitarism is believed to be due to a deficiency of anterior pituitary and target hormones (9). Many hormonal deficiencies, such as hypothyroidism, adrenal insufficiency, and gonadal hormonal deficiency, can explain normochromic anemia in hypopituitarism (11). It can also result from a physiologic adjustment to lower oxygen requirement, as pituitary hormones modulate the production of erythropoietin in the kidney (8, 9, 10).

The pathogenesis of pituitary failure following PPH is not clear. Sheehan & Davies (1) proposed vasospasm of the pituitary vessels and consequent ischemic necrosis of the pituitary. However, experimental evidence suggests efficient adaptive autoregulation of adenohypophyseal blood flow during hypotension caused by acute blood loss. Hormonal deficiencies, such as hypothyroidism, adrenal insufficiency, and gonadal hormonal deficiency, can explain the existence of normochromic anemia in hypopituitarism patients. The anemia associated with anterior pituitary hormonal deficiencies shows a normal red cell lifespan. The anterior hypopituitarism may be related to either a normal bone marrow or a deficiency in erythropoiesis. Despite the rarity of pancytopenia as a manifestation of hormone deficiency, several hematologic features in this case suggest that the cause of pancytopenia is due to hormonal deficiency. Only few cases have been reported in which pancytopenia originated from endocrine dysfunctions. Pure substitution therapy with peripheral hormones resulted in a correction of the hematological abnormalities in peripheral blood and bone marrow (2, 4, 8). Interestingly, there was a published case about a 60-year-old man who presented with progressive exhaustion and polyuria, and whose laboratory results discovered a lack of the hormones related to the action of the anterior pituitary gland (testosterone, thyroid hormone, prolactin, cortisol, and insulin-like growth factor-1). Imaging of the pituitary established a cystic mass consistent with a pituitary adenoma, substituting a huge part of the normal pituitary tissue (11). In addition, it is described that severe hepatic steatosis is highly prevalent in SS. Das *et al.* concluded in their study that subclinical cardiac systolic dysfunction is common in such patients, but of mild intensity (12).

Our patient was admitted to the Department of Endocrinology after a hematological examination for morphological and functional examination of the hypophyses. In the MRI, the patient’s pituitary gland was hypoplastic. Sella turcica was filled with the cerebrospinal fluid (empty sella). Analyzing the basal hormone secretions and stimulation tests (ACTH test and insulin tolerance test), we diagnosed subsequent panhypopituitarism (secondary hypercorticism, hypothyreosis, hypogonadism, and growth hormone deficiency (GHD) in adults). According to the fact that there was a massive hemorrhage followed by the shock after the third delivery, we concluded that it was SS. After diagnosis, the patient was started on replacement of hydrocortisone and thyroxine. A reevaluation including a complete medical history, physical examination, and biochemical reassessment was done after 5 months of treatment. The patient’s symptoms, such as general weakness and slow speech, improved remarkably. Her body weight increased from 39.0 to 49.0 kg. The free T4 level was normal. Complete hematological recovery was seen after making the patient eucortisolemic and euthyroid: the leukocyte count, the hemoglobin level, and the platelet count were all within the normal range.

In the present study, we described a patient who survived acute blood loss after the third delivery, followed by related pituitary failure for several years without any hormone replacement. Despite lactational failure and amenorrhea dating from the postpartum period, the pituitary insufficiency was not suspected by the physicians until life-threatening symptoms of circulatory collapsed. This loss of pituitary trophic hormone functions suggests that the destruction of the pituitary gland was partial to progressive loss over time, especially thyrotroph and corticotroph function. Such delayed presentation of SS could be due to the subtlety of symptomatology, and thus associated failure to recognize hypopituitarism.

The mechanisms of pancytopenia and the hypoplastic bone marrow in anterior hormone deficiency have not been investigated. In addition, even the clinical aspects of pancytopenia and the hypoplastic marrow induced by panhypopituitarism are unclear. In a patient with pancytopenia in the presence of panhypopituitarism, hormone replacement should be undertaken promptly. The case series of SS and pancytopenia described by Laway *et al.* (8) suggested that thyroid and cortisol hormone replacement are ultimately responsible for bone marrow recovery.

As multiple hormone deficiencies in SS are often replaced simultaneously, it is difficult to discern if the hematological abnormalities are related to any one or all of the deficient hormones and can be responsible for pancytopenia (13). Early diagnosis and appropriate treatment are important to reduce the morbidity and mortality of patients with SS (14). The goal of therapy is to replace deficient hormones, not only to correct endocrine abnormalities but also to reduce mortality due to hypopituitarism (15). SS is a frequent cause of hypopituitarism, but the clinical features are often subtle and years may pass before the diagnosis is made (15).

In summary, we report our experience of a cure for pancytopenia originating from hypopituitarism after corticosteroid and thyroid hormone replacement therapy. Clinicians should consider the possibility of hypopituitarism as a cause of pancytopenia and indicate a series of hormonal examinations.

## Declaration of interest

The authors declare that there is no conflict of interest that could be perceived as prejudicing the impartiality of the work reported.

## Funding

This work did not receive any specific grant from any funding agency in the public, commercial, or not-for-profit sector.

## Patient consent

Written informed consent was obtained from our patient for the publication of this case report.

## Author contribution statement

VM cared and treated the patient. RZZ, JN, SS, DB, ZI, NI, and PD wrote the manuscript.
